# Dr. Stricker on the Hospitals of Italy

**Published:** 1842-12-03

**Authors:** 


*The Hospitals of Italy.* By Dr. Stricker.—The Universities of Padua and Pavia are arranged entirely on the Austrian footing; in the others all the lectures are gratuitous. There is but one course in the year; the vacation consists of July, August, September, and, by an abuse, of October also. To this must be added the numerous holidays, so that the number of lecture-days is often very limited; in Lucca, for example, only 143. Hence the time of study is continued pretty long: in the States of the Church, for instance, there is a philosophical course lasting two years, with an examination; then three years' study for surgeons, and four for physicians; and, lastly, two years' practice in the *clinique*, after taking their degree, before admission to the state examination. The practice of physic is more or less governed by three different systems; first, by Rasori's doctrine of contra-stimulus, of which Tommasini is the chief support; by Bufalini's system, who tries to explain diseases by organico-chemical disturbances; and by Pucinotti's, who endeavours to explain them by magnetico-electric disturbances; though the theories of the two last have but little influence on their practice. The treatment is on the whole simple, with much reference to diet: new remedies are admitted with difficulty, so that the *oleum jecoris aselli* (cod's liver oil) is not yet known in southern Italy. Homœopathy has found but few adherents, and hydropathy none. The *cliniques* are partly in the French style, as at Naples and Rome, where they are nothing more than lectures; and partly after the German manner, and at the bedside of the patient. *Polycliniques* [*i. e.* dispensary visits, together with pupils, at the patients' homes] are not usual.

In surgery and midwifery there is a great inclination to the practice of the French, whose principal works are translated into Italian, and whose instruments are used by preference. In the treatment of eye diseases they follow German authors more.

*Naples.*—In Naples there are three institutions for the study of medicine.

1. The University.

2. The Medical College in the neighbourhood of the *Incurabili*, for 120 young persons, who pay ten ducats a month. They have dormitories, a garden, and a public refectory; are present at the university lectures, and afterwards become assistants at the *Incurabili*.

3. The professors not yet appointed to chairs receive fees, for which they give lectures and hold a *clinique*. To the last belongs the medical *clinique* of Dr. Prudente, which he holds at the patient's bed-side, in the hospital of the *Madonna di Loreto*. Prudente also gives lectures on anatomy in the small lecture-room of his hospital. This hospital contains a collection founded by Pietro Sorrentino, which extends over the domain of mineralogy, botany, zoology, and anatomy. It possesses many wax preparations, *e. g.* the aurantiaceæ, the mollusca (which, however, have their natural shells), the diseases of the eye and the skin, and the varieties of the arteries according to Tiedemann. Giampetri is the director of the surgical division.

The principal civil hospital is the *Ospedale degl' Incurabili*, in an open situation on a small hill at the limits of the old town. It is capable of holding 2,500 patients, and has a revenue of more than 100,000 ducats. It contains the university *cliniques*, the medical one, and the surgical one under de Horatiis, where the representations of interesting cases, which are suspended on the walls, cannot be very cheering to the patients; the eye *clinique*, which, since 1821, had been under Quadri; and the obstetric one under Cattolica. The last has 45 beds for pregnant women, two beds for delivery, and an apartment for lying-in women. Besides the wards for patients, which are very large, and built in the form of a cross, the building contains a room for lithotomy; a ward for mercurial frictions; and one which serves as an *hospice* for old incurable ones; in addition to lecture-rooms, an anatomical theatre, a small *clinique* for examination, and an apartment for patients who pay 12 or 18 ducats a month.

Patients who want to be taken in, assemble in the morning in two wards, and are then selected.

The *Albergo dei Poveri*, also called *Reclusorio*, or *Seraglio*, the great building at the end of the *Strada Foria*, was originally intended to be a square, but the front alone has been completed. This enormous poor-house, founded in 1742, contains 2,700 men and boys, mostly foundlings, who wear a uniform, and 700 women. In the lower rooms are workshops of different kinds; glass furnaces, a type foundry, a pin manufactory, a forge for arms, &c. The dormitories are airy, and contain from 56 to 128 beds each. The diet consists of bread, soup, macaroni, and wine for dinner, and soup, with bread, for supper; and three ounces of meat on Sundays, Thursdays, and holidays.

In the same building is the institution for the deaf and dumb, containing 32 pupils under the care of Cozzolino, who are instructed in religion, reading, writing, arithmetic, geography, and natural history. The patients of the *Albergo* go to the hospital of *St. Madonna di Loreto.*

Close by is the Botanic Garden, whose director is Tenore, and whose gardener is Denhardt, a Hanoverian. It contains a beautiful *tepidarium* of the Doric order, entirely covered with *Ficus stipulata:* the *caldarium* contains nothing but coffee-trees. The arrangement of the aquatic plants is bad. The plants are arranged in four squares according to Linnæus, and in two according to Jussieu.

The institution for the blind, *St. Giuseppe e Lucia*, on the Chiaja, is arranged after the pattern of the best institutions of the kind. One hundred and sixty blind persons are instructed in reading, music, mathematics, and geography: and they print their books themselves with letters in relief. There is among them an interesting case of *elephantiasis leonina.* The Director is Professor Quadri.

The Naval Hospital is also on the Chiaja; the best wards, which are on the first floor, are occupied by the marines; those on the second, by patients from the galleys.

In front of the Capuan Gate, in the Hospital of Prisons (*San Francesco*) is the anatomical, zoological, and pathological cabinet collected by Nanula, formerly Professor of Anatomy. It contains many most remarkable specimens. For example, there is a specimen of quintuplets, four female and one male; they were born at 6¾ months, and one lived a quarter of an hour, another half an hour. There is also an ossified ovary weighing 9½ pounds. The collection, which is elegant, but ill arranged has been bought by the Government, and after the decease of the present prosessor is to be transferred to the University. Near it is the hospital called *Sta. Maria della Fede*, for girls of the town; the Director is Giampietri. There is an examination every Wednesday; when cured they are kept a week in the house and observed. The sores are touched with nitrate of silver. When the chief disease is in the glands, the general treatment consists of [mercurial] frictions; when the bones principally are affected, of Dupuytren's sublimate pills. According to the principles which prevail here, the patients have nourishing diet; eleven ounces of bread, with three of meat, four of macaroni, and a tumbler of wine, for dinner; soup, with six ounces of bread, for supper. Schultz's work on the mineral springs of Naples (Berlin, 1837) may be consulted for information respecting the baths of Castellamare and Ischia; observing, however, that the baths of *Torre dell' Annunziata* have already failed.—*London Medical Gazette*, October 21, 1842.

